# A MYB-related transcription factor ZmMYBR29 is involved in grain filling

**DOI:** 10.1186/s12870-024-05163-9

**Published:** 2024-05-27

**Authors:** Jia Wen Wu, Xiao Yi Wang, Ru Yu Yan, Guang Ming Zheng, Lin Zhang, Yu Wang, Ya Jie Zhao, Bo Hui Wang, Meng Lin Pu, Xian Sheng Zhang, Xiang Yu Zhao

**Affiliations:** State Key Laboratory of Crop Biology, College of Life Sciences, Shandong Agricultural University, Taian, 271018 Shandong China

**Keywords:** Basal endosperm transfer layer (BETL), Endosperm, MYB-related transcription factor, Maize seed

## Abstract

**Background:**

The endosperm serves as the primary source of nutrients for maize (*Zea mays* L.) kernel embryo development and germination. Positioned at the base of the endosperm, the transfer cells (TCs) of the basal endosperm transfer layer (BETL) generate cell wall ingrowths, which enhance the connectivity between the maternal plant and the developing kernels. These TCs play a crucial role in nutrient transport and defense against pathogens. The molecular mechanism underlying BETL development in maize remains unraveled.

**Results:**

This study demonstrated that the MYB-related transcription factor ZmMYBR29, exhibited specific expression in the basal cellularized endosperm, as evidenced by in situ hybridization analysis. Utilizing the CRISPR/Cas9 system, we successfully generated a loss-of-function homozygous *zmmybr29* mutant, which presented with smaller kernel size. Observation of histological sections revealed abnormal development and disrupted morphology of the cell wall ingrowths in the BETL. The average grain filling rate decreased significantly by 26.7% in *zmmybr29* mutant in comparison to the wild type, which impacted the dry matter accumulation within the kernels and ultimately led to a decrease in grain weight. Analysis of RNA-seq data revealed downregulated expression of genes associated with starch synthesis and carbohydrate metabolism in the mutant. Furthermore, transcriptomic profiling identified 23 genes that expressed specifically in BETL, and the majority of these genes exhibited altered expression patterns in *zmmybr29* mutant.

**Conclusions:**

In summary, *ZmMYBR29* encodes a MYB-related transcription factor that is expressed specifically in BETL, resulting in the downregulation of genes associated with kernel development. Furthermore, ZmMYBR29 influences kernels weight by affecting the grain filling rate, providing a new perspective for the complementation of the molecular regulatory network in maize endosperm development.

**Supplementary Information:**

The online version contains supplementary material available at 10.1186/s12870-024-05163-9.

## Background

The endosperm of maize serves as a strong sink for nutrients loaded from the maternal plant, supplying a substantial amount of raw materials for human food, animal feed, and biofuels [1]. Elucidating the mechanisms underlying maize endosperm development is crucial for enhancing food security [2]. Comprising approximately 80-85% of the total mature grain’s weight, the endosperm provides nutrition for embryo development and germination. The triploid endosperm development is initiated by double fertilization and progresses through four distinct phases: coenocyte, cellularization, cell differentiation, and the stages of filling and dry matter accumulation [3–5]. After undergoing cell proliferation and cell differentiation, the endosperm is structured into four distinct regions: aleurone layer (AL), embryo surrounding region (ESR), starchy endosperm (SE) and basal endosperm transfer layer (BETL) [6–8]. As a permanent tissue with a large volume and diverse cell types, compared to other crops, the maize endosperm is an excellent model for dissecting kernel development patterns [9].

The BETL, located at the base of the endosperm, serves as a critical junction between the maternal tissue and the developing seed, playing a pivotal role in grain filling and defense processes. In maize, nutrient transfer from material tissue to the filial kernel compartments occurs across the placento-chalazal zone (PC), and the maternal cells in this region undergo a series of programmed cell deaths [10, 11]. The BETL is characterized by its rounded, arched structure, composed of two to three layers of highly specialized, elongated cells [12]. During early development, the transfer cells (TCs) within the BETL undergo specialized wall ingrowths, generating a distinct structure known as cell wall ingrowths (CWIs). The CWIs exhibit a sponge-like network structure and possess a thickened cell wall [7, 13–15]. These CWIs facilitate the efficient translocation of essential nutrients, including sugars, inorganic salts, and water, from the maternal tissue to the endosperm. The BETL is the sole conduit for nutrient transfer between the maternal plant and filial kernel compartments, as the entire endosperm is surrounded by the coat layer, which comprises the cuticle and pericarp [16, 17]. Sucrose and hexoses enter the TCs from the PC region posterior to the phloem. The cell wall invertase 2 (*ZmINCW2*) is responsible for converting sucrose released from the maternal tissue into hexoses. In the *mn1* mutant, endosperm transfer cells lose their typical cell wall ingrowth characteristics, resulting in significantly decreased glucose and fructose in the endosperm and increased sucrose levels [18, 19]. The BETL-specific gene *ZmSWEET4c*, encoding a sugar transporter, is primarily responsible for transporting hexoses into the grain. Mutation of *ZmSWEET4c* leads to reduced grain size and the endosperm transfer cells exhibit a loss of their characteristic wall ingrowth features [20, 21]. Interestingly, sucrose also acts as a signaling molecule to induce BETL differentiation, further confirming that the morphogenesis of the BETL is intricately linked to its function.

Both laser-capture microdissection data and a single-cell transcriptomics profile demonstrate the complexity of the regulatory network underlying BETL analysis, and there are still numerous mysteries to be unraveled in this field [22, 23]. The MYB (v-myb avian myeloblastosis viral oncogene homolog) transcription factor family, identified in 1987, is one of the most prominent transcription factor families in plants and plays a pivotal role in regulating various biological processes [24–27]. The MYB transcription factor family can be divided into several subfamilies. Despite numerous studies on the R2R3-MYB gene family, there are few reports on the functions of MYB-related genes. The MYB-related transcription factor, which contains the MYB/SANT domain, is a subclass of the MYB family and is widely distributed in plants [28–32]. Previous studies have shown that the MYB transcription factor family primarily regulates stress and abiotic stress responses by participating in redox reactions and hormone synthesis [33–35]. In addition, MYB transcription factors are also involved in the regulation of the circadian rhythm [36, 37], the development of trichomes and root hairs [38, 39], and flowering time [40]. The MYB-related transcription factor participates in the TCs morphogenesis by acting on genes expressed specifically in BETL; however, the specific mechanism requires further research. *ZmMRP1*, which encodes a MYB transcription factor containing a DNA-binding domain, has been identified as a key regulator of transfer cell differentiation and the endosperm development. Ectopic expression of *ZmMRP1* can generate CWIs in AL, providing evidence for its critical role in establishing BETL morphology [18, 41]. *ZmMRP1* can activate the transcription of multiple BETL-specific expressed genes, indicating that it is a key regulatory factor for BETL [15, 16, 42, 43].

We identified a BETL-specific expressed transcription factor, ZmMYBR29, which possesses a conserved SANT/MYB-like domain and belongs to the MYB-related transcription factor family. This gene has a close evolutionary relationship with the key factor ZmMRP1, which regulates the BETLs differentiation. *ZmMYBR29* commences expression in kernels at 4 days after pollination (DAP) and expresses specifically in the BETL. In the homozygous loss-of-function *zmmybr29* mutant, the TCs in BETL exhibited abnormal morphology, leading to a decreased grain filling rate. *ZmMYBR29* is implicated in the regulation of biological pathways associated with kernel development, including starch synthesis and carbohydrate metabolism. Additionally, the expression levels of 23 genes expressed specifically in BETL were altered in *zmmybr29* mutant. In conclusion, *ZmMYBR29* affects maize kernel size by participating in maize endosperm development.

## Results

### Identification of a MYB-related transcription factor ZmMYBR29 in maize kernel

**Fig. 1:**
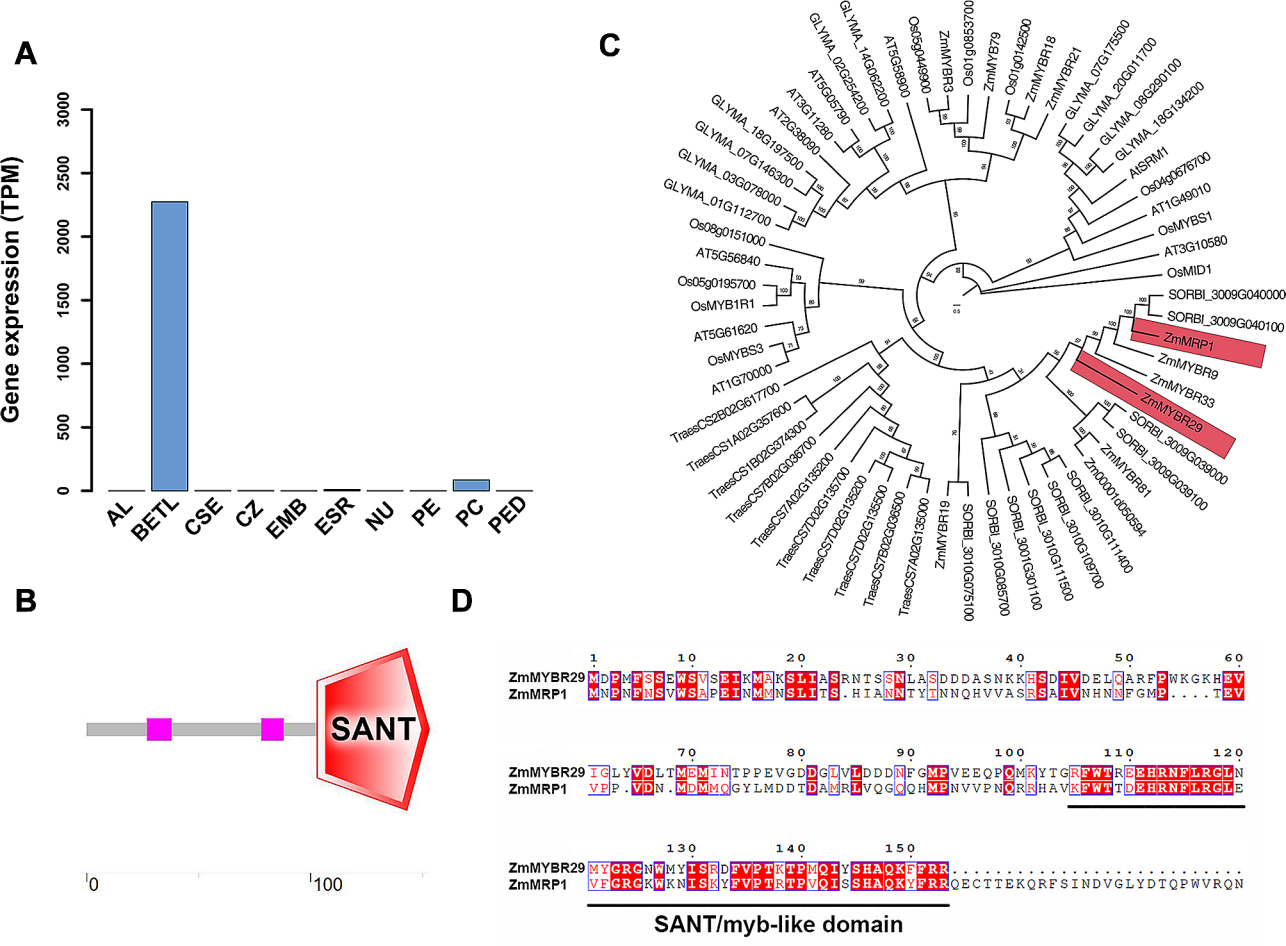
ZmMYBR29 and ZmMRP1 are homologous. **A** Spatial expression pattern analysis of ZmMYBR29. AL, aleurone; BETL, basal endosperm transfer layer; CSE, central starchy endosperm; CZ, conducting zone; EMB, embryo ESR embryo surrounding region; NU, nucellus; PE, pericarp; PC, placenta chalaza; PED, pedicel. **B** Analysis of the SMART protein database predicted that the amino acid region from position 103 to 153 in ZmMYBR29 is a SANT/MYB-like domain. **C** Phylogenetic analysis of ZmMYBR29 in *Arabidopsis*, rice, soybean, wheat, and maize. The numbers on the branches indicate the reliability percent of bootstrap value based on 1000 replications, and the scale bar represents 0. 5 substitutions per amino acid. **D** Amino acid sequence alignment of ZmMYBR29 and ZmMRP1

In maize (*Zea mays* L.), the endosperm as a permanent tissue can accumulate nutrients for embryo development and germination. Histological examination of developing endosperms has revealed that the basal endosperm transfer layer (BETL) contains the cell wall ingrowths (CWIs), which primarily mediate nutrient transport from the maternal tissue. The advent of next-generation sequencing (NGS) technology has enabled the construction of high-resolution gene regulation maps. Utilizing laser-capture microdissection data from 8 days after pollination (DAP) maize kernels reported in 2015, we have identified several genes that encode transcription factors, including ZmNYBR29, which are expressed explicitly in the BETL. *ZmMYBR29* expressed specifically in the BETL of differentiated endosperm [22] (Fig. 1A; Additional file 2: Table S1). A recent study utilizing single-cell resolution dynamic expression mapping has confirmed the BETL-specific expression of *ZmMYBR29* in maize [23].

*ZmMYBR29* encodes a MYB transcription factor. Protein domain prediction analysis using SMART revealed a conserved SANT/MYB-like domain (103–153 aa) at the C-terminal of the protein, which is highly similar to the MYB DNA binding domain (Fig. 1B). Based on this finding, we hypothesized that ZmMYBR29 is a member of the MYB-related transcription factor family.

To investigate the biological function of ZmMYBR29 and confirm its classification within the MYB-related transcription factor family, a phylogenetic analysis was conducted. BLAST search identified 60 homologous proteins. The NCBI database was used to search homologous proteins in the dicot *Arabidopsis* (*Arabidopsis thaliana*), soybean (*Glycine max* (L.) Merr.) and monocots such as maize, rice (*Oryza sativa*) and wheat (*Triticum aestivum*). Maximum likelihood phylogenetic analysis revealed that ZmMYBR29 belongs to the MYB-related transcription factor family and exhibits high homology with several family members, including ZmMYBR9 and ZmMYBR33 (Fig. 1C). Notably, ZmMYBR29 exhibits homology to ZmMRP1, a key transcription factor involved in specific morphogenesis of transfer cells (TCs) in the BETL [18]. Both ZmMYBR29 and ZmMRP1 contain a conserved SANT/MYB-like domain (Fig. 1D).

In summary, we identified a BETL-specific expressed gene *ZmMYBR29* through transcriptome analysis, which encodes a MYB-related transcription factor with significant homology to ZmMRP1. It is considered a potential regulator of TCs morphogenesis.

### *ZmMYBR29* expressed specifically in the maize kernel BETL

**Fig. 2:**
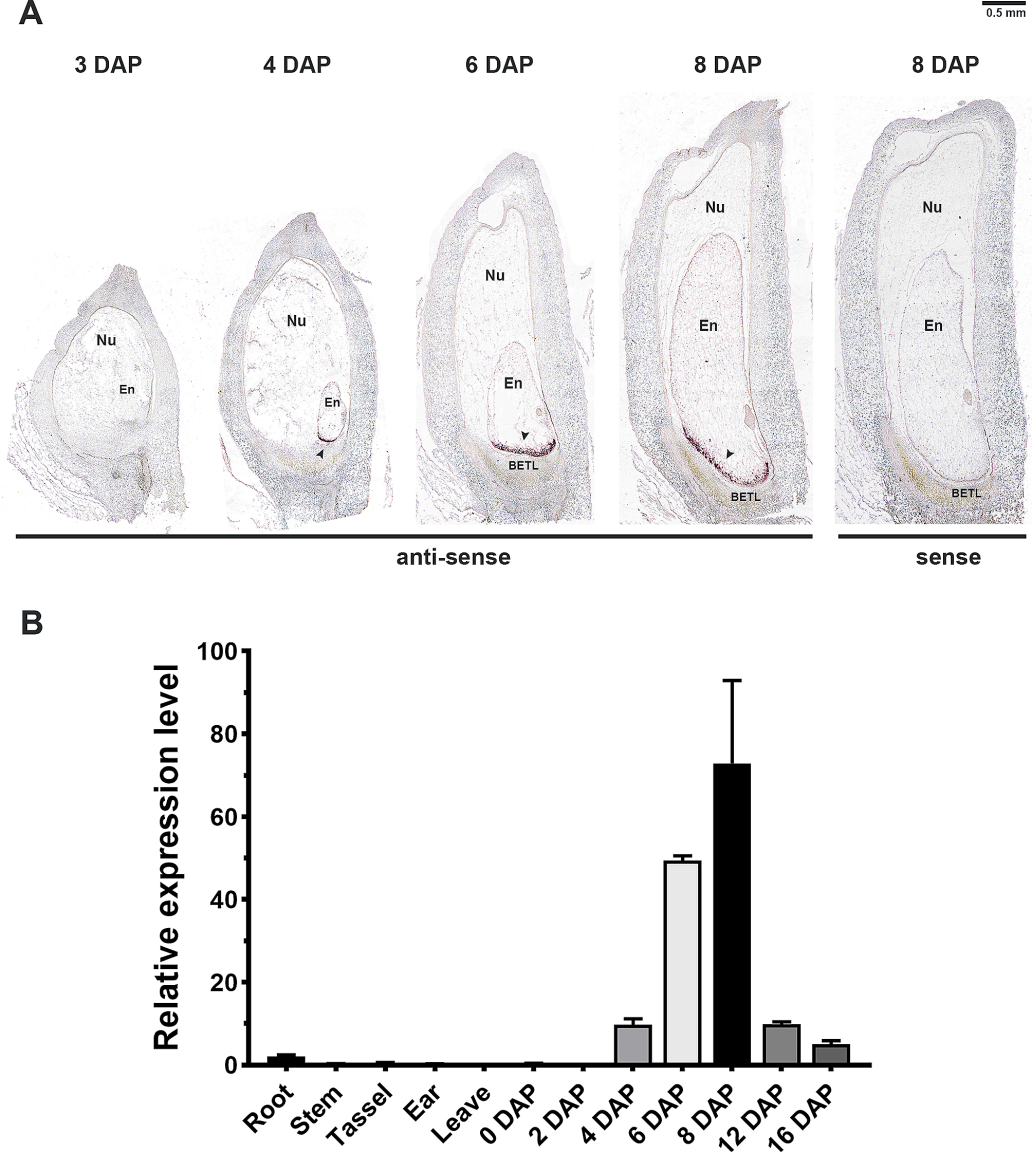
*ZmMYBR29* expressed specifically in the BETL. **A** In situ hybridization of *ZmMYBR29*. Histological sections of maize kernels at 3, 4, 6 and 8 DAP were hybridized with antisense and sense. DAP, days after pollination; anti-sense: antisense probe; sense, sense probe serves as a control; Nu, nucellus; En, endosperm; BETL, basal endosperm transfer layer; Scale bars, 0.5 mm. **B** Real-time PCR detection of *ZmMYBR29* expression levels in various tissues of maize

To elucidate whether *ZmMYBR29* exhibits a specific expression pattern during maize kernel development, in situ hybridization experiment were conducted to verify the spatial and temporal expression of *ZmMYBR29* in maize kernels. The expression pattern of *ZmMYBR29* were examined at 3, 4, 6 and 8 DAP in the inbred line B73 (Fig. 2A). Following double fertilization, the endosperm undergoes cellularization, which determines the cell number at 3–4 DAP, and the endosperm cells differentiate into mature TCs at 6–8 DAP. Consequently, the expression pattern of ZmMYBR29 was analyzed at these two critical developmental stages to discern its regulatory role.

We found that *ZmMYBR29* expressed specifically in the basal cellularized endosperm compared to the control, with a distinct signal detectable at the endosperm cellularization stage at 4 DAP (Additional file 1: Fig. S1). *ZmMYBR29* was expressed at the basal of cellularized endosperm. adjacent to the placenta-chalazal region, as indicated by the black arrow. This observation suggested that *ZmMYBR29* functions early in the endosperm differentiation and serves as a potential regulatory factor for BETL differentiation and morphogenesis.

Moreover, qRT-PCR experiments were performed to assess the expression level of *ZmMYBR29* in various tissues of maize (Fig. 2B). These results indicated that *ZmMYBR29* exhibited a specific expression pattern in the endosperm, beginning at 4 DAP and reaching its peak expression level at 8 DAP during the endosperm differentiation stage. Following the completion of endosperm differentiation and the initiation of grain filling, the expression level of *ZmMYBR29* progressively decreases. Additionally, the expression of *ZmMYBR29* was analyzed using the published transcriptome data of kernels at different hours after pollination [6]. The data analysis indicated that *ZmMYBR29* began to express at about 84 h after pollination, corroborating the results of the in situ hybridization study [22](Additional file 1: Fig. S2).

These findings collectively suggest that *ZmMYBR29* is expressed exclusively in the basal cellularized endosperm at 4 DAP hinting at its potential role as a transcription factor that triggers the TCs morphogenesis.

### *ZmMYBR29* is involved in regulating grain size

**Fig. 3:**
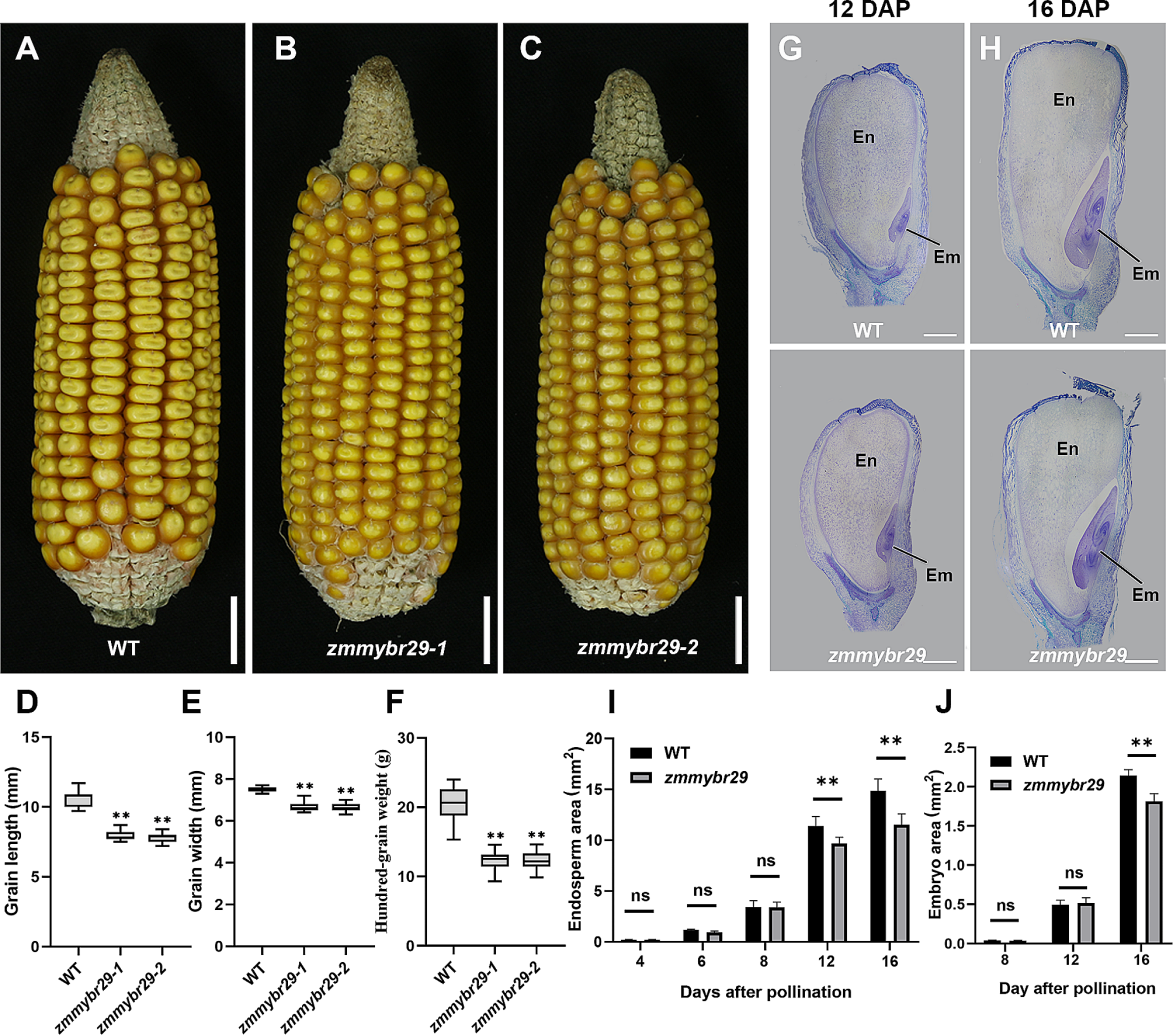
The homozygous loss-of-function mutant *zmmybr29* led to small kernels. **A**-**C** Phenotype of the ears of the WT and *zmmybr29*. Scale bars in **A**-**C** are 1.5 cm. **D** Grain length of mature WT and *zmmybr29* kernels. **E** Grain width of mature WT and *zmmybr29* kernels. **F** 100-grain weight of mature WT and *zmmybr29* kernels. **G**-**H** Histological sections of WT and *zmmybr29* kernels of 12 DAP and 16 DAP. Nu, nucellus; En, endosperm. Em, embryo. Scale bars, 1 mm. The upper panel of G-H is histological sections of WT; The lower panels of **G**-**H** is histological section of *zmmybr29*. **I**-**J** Area statistics of endosperm and embryo of WT and *zmmybr29* kernels. Error bars indicate the standard deviation (SD). **, *P* < 0.01; ns, no significance (Student’s *t*-test)

We constructed loss-of-function mutants via CRISPR/Cas9 to confirm that *ZmMYBR29* has a role in the BETL morphogenesis. The guide RNA was designed on the first exon (Additional file 1: Fig. S3A, B). Through multiple generations of self-crossing, we obtained two stable genetic transgenic lines, *zmmybr29-*1 and *zmmybr29-*2, and the background was further purified via multigeneration backcrossing with B104. In order to screen for lines with vector segregation and editing in the T_1_ transgenic population, we extracted the genomic DNA from each T_1_ plant and performed PCR amplification to identify whether the CRISPR/Cas9 vector exists and sequencing to detect whether the *ZmMYBR29* was edited. After sequencing, we found that there was a single base deletion at the second target site in *zmmybr29*-1, resulting in a frameshift mutation. Additionally, *zmmybr29*-2 exhibited a deletion of 4 bases at the first target site and 2 bases at the second target site, also leading to a frameshift mutation (Additional file 1: Fig. S3C). Subsequently, these T_1_ heterozygous transgenic plants were self-crossed, and subsequent sequencing screening was performed to select homozygous mutants with reduced grain size in the T_2_ generation for further study. Sequencing analysis revealed that the frameshift mutation in both lines was caused by the deletion of bases, which interrupted protein functional domains (Additional file 1: Fig. S3D). Both lines exhibited a developmental defect in kernel formation, with kernels that were substantially smaller than those of the wild type (Fig. 3A-C). Compared to the WT, the mutant displayed a significant decrease in grain length and width, the 100-grain weight decreased by 38% in *zmmybr29*, while no significant differences in grain thickness were observed (Fig. 3D-F; Additional file 1: Fig. S4; Additional file 3: Table S2). Additionally, no significant variations in plant height were observed between mutants and the WT (Additional file 1: Fig. S5), suggesting that ZmMYBR29 is involved specifically in the regulation of grain size.

Paraffin sections of *zmmybr29-*1 were stained with toluidine blue O to study kernels development between 4 and 16 DAP from a cytological perspective (Additional file 1: Fig. S6). Compared with those of the WT, there was no significant difference in grain size at the early stage between 4 and 12 DAP, and the morphology of the embryo and endosperm appeared intact. Both embryo and endosperm development were delayed at 12–16 DAP (Fig. 3G, H). Image J software was utilized to analyze the endosperm and embryo areas (Fig. 3I, J). The data analysis revealed a significant reduction in endosperm area at 12 DAP and a notable decrease in embryo area at 16 DAP in the mutant.

This observation suggested that *ZmMYBR29* plays a crucial role in grain size regulation, and its disruption leads to defective embryo and endosperm development.

### *ZmMYBR29* is essential for the BETL morphogenesis and function

**Fig. 4:**
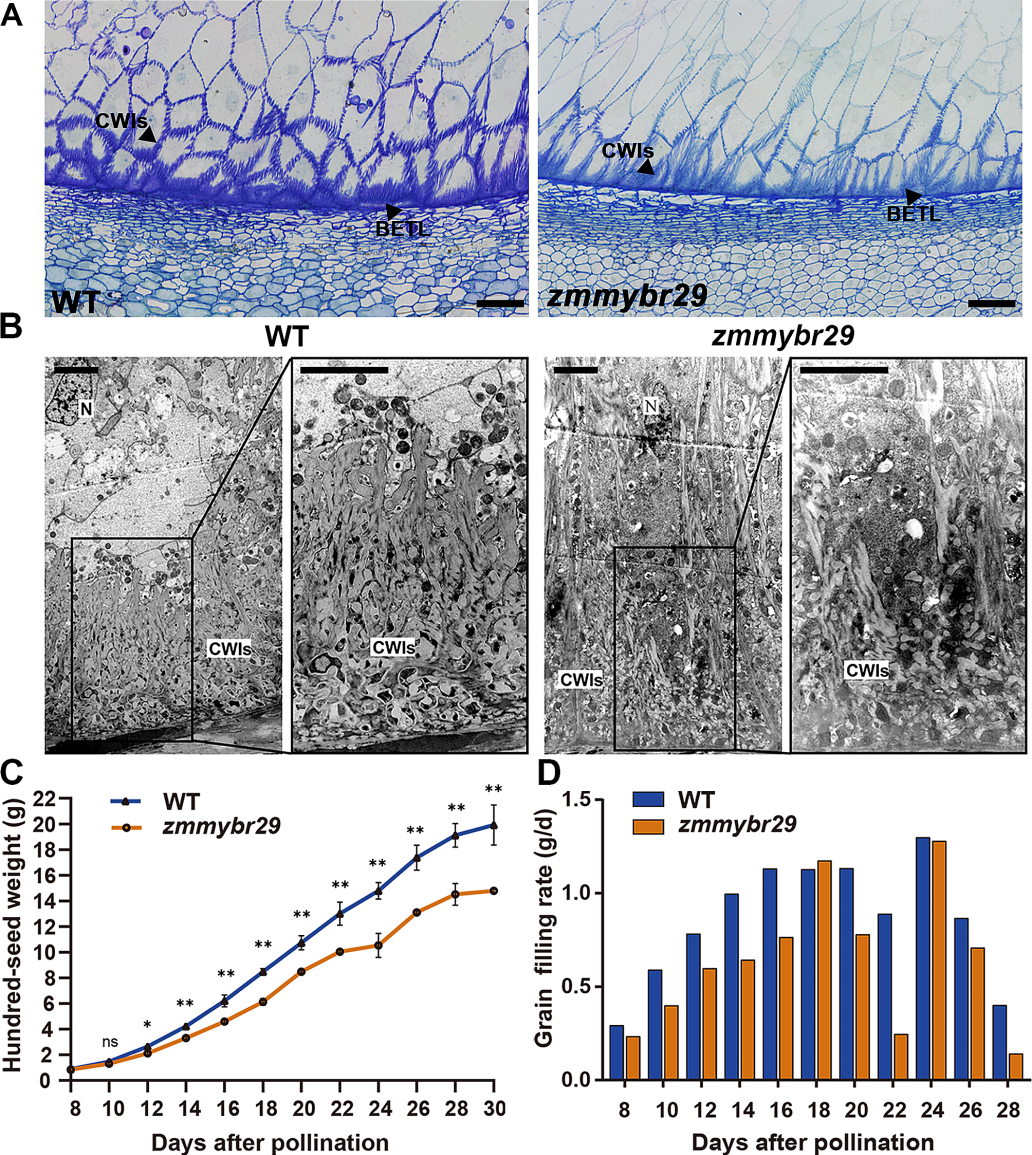
*zmmybr29* leads to abnormal morphogenesis and function of BETL cells. **A** Semithin sections of WT and *zmmybr29* kernels. Semithin sections stained with 0.1% TBO for complete TC layer of 18 DAP kernels from both the WT and *zmmybr29* mutant. Scale bars, 50 μm. **B** Transmission electron microscopy observation of the BETL between WT and *zmmybr29* kernels. BETL, basal endosperm transfer layer; CWIs, cell wall ingrowths; Nu, nucleus. Scale bars, 5 μm. **C** 100-grain weight of WT and *zmmybr29* kernels at 8–30 DAP. The blue line represents WT and the orange line represents *zmmybr29*. The error bars indicate SD. **, *P* < 0.01; *, *P* < 0.05; ns, no significance (Student’s *t*-test). **D** Comparison of the grain filling rates of WT and *zmmybr29* kernels at 8–30 DAP. The blue box represents the WT and the orange box represents *zmmybr29*

To determine the effects of *ZmMYBR29*, which expressed specifically in the BETL, on TCs morphogenesis and BETL function, we further investigated the micromorphology of the BETL by using semithin sections (Fig. 4A). In the WT, the CWIs were dense and evenly distributed within the BETL, whereas in the *zmmybr29* mutant, the CWIs were narrowly distributed. Utilizing transmission electron microscopy (TEM) to observe BETL single cells at 18 DAP, we observed a reduction in the number of CWIs in the *zmmybr29* mutant compared to the WT, with the remaining CWIs exhibiting a sparse distribution and abnormal morphology (Fig. 4B). This indicated that the loss of function in *ZmMY*BR29 results in abnormal TCs morphology.

To validate the impact of *ZmMYBR29* on BETL function further, we assessed the grain filling rates in WT and *zmmybr29*. Kernels were peeled at 8–30 DAP, with three biological replicates collected every two days from the middle of the ear and dried. Subsequently, the 100-grain weight was measured and calculated (Fig. 4C). No significant difference in 100-grain weight was detected between WT and *zmmybr29* at 8–10 DAP. However, the 100-grain weight in *zmmybr29* progressively decreased from around 12 DAP. Beginning at 12 DAP, the increase in the grain weight of *zmmybr29* was slower than WT. At 10 DAP, the grains began to accumulate starch and dry matter. At this time, the differences in 100-grain weight indicate an abnormal loading function of BETL.

Statistical analysis of the grain filling rate showed that although the overall trends of filling were consistent for WT and *zmmybr29*, the grain filling rate of *zmmybr29* was significantly lower than that of WT from 8 to 30 DAP (Fig. 4D). The average grain filling rates were 0.86 g/d for WT and 0.63 g/d for *zmmybr29*, with *zmmybr29* exhibiting a 26.7% reduction in grain filling rate compared to WT (Additional file 4: Table S3). This confirmed that the reduced grain filling rate in *zmmybr29* leads to a decrease in dry matter accumulation, which ultimately results in a reduction in grain weight.

In short, *ZmMYBR29* is vital for the maintenance of the morphological integrity and biological function of BETL, the narrowed range of CWIs and the abnormal accumulation of dry matter in grains were observed in *zmmybr29.*

### Abnormal expression of genes related to maize kernel development in *zmmybr29*

To explore the biological pathways and genes directly or indirectly regulated by *ZmMYBR29*, we performed RNA sequencing (RNA-seq) on kernels from WT and *zmmybr29* at 12 DAP. We identified the differentially expressed genes (DEGs). Principal component analysis of the dataset indicated high reproducibility among the three biological replicates (Additional file 1: Fig. S7A, B). A total of 405 significant DEGs were detected in the *zmmybr29* vs. WT, with 164 genes upregulated and 241 genes downregulated in *zmmybr29* (TPM>1, Log2FC±0.42, FDR<0.05, Additional file 5: Table S4).

**Fig. 5:**
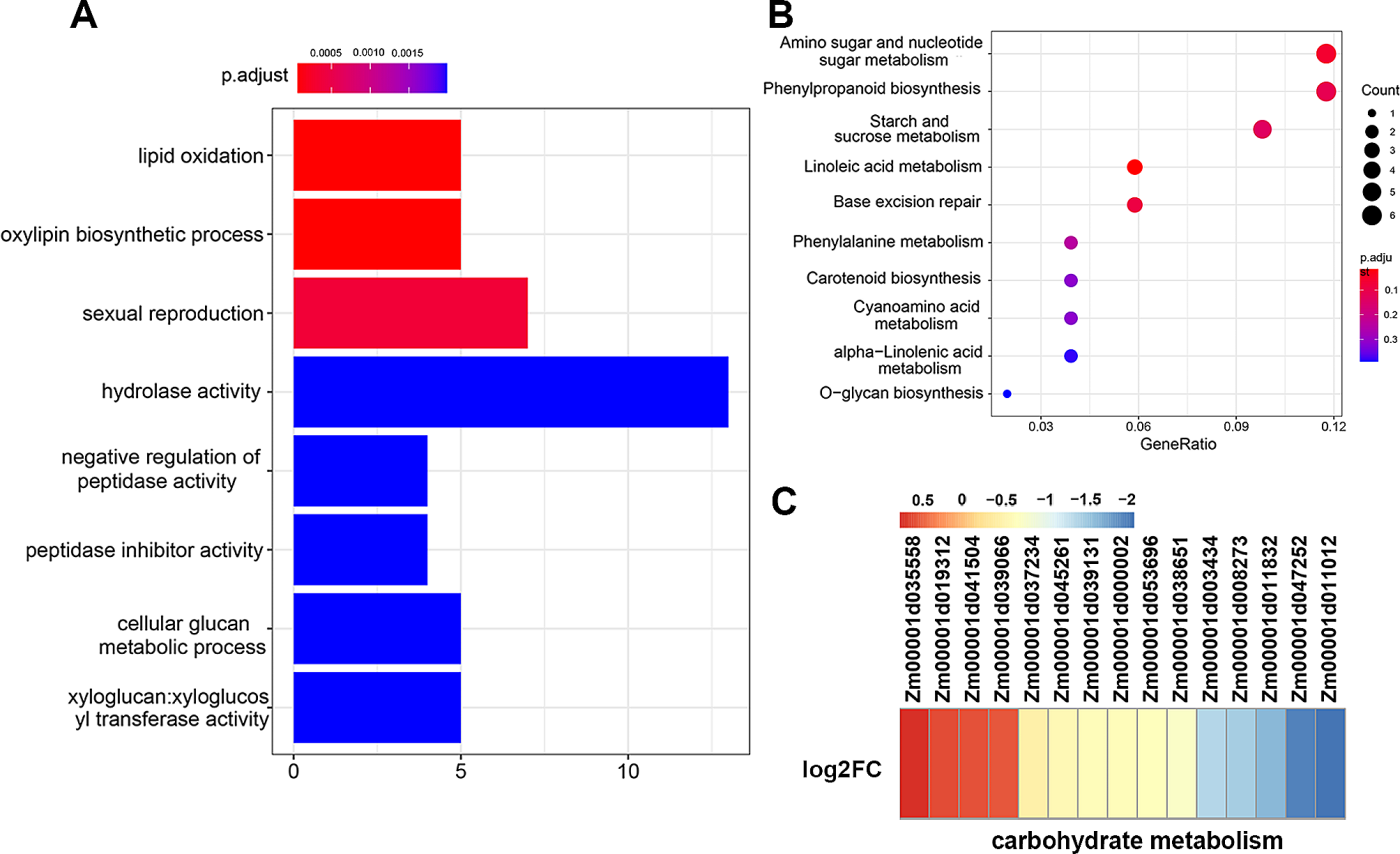
*ZmMYBR29* regulates the expression of genes related to kernels development. **A** GO enrichment analysis of DEGs. **B** KEGG analysis of DEGs. **C** Gene expression heatmap related to carbohydrate metabolism

Gene ontology (GO) analysis indicated that the DEGs were primarily clustered within five major biological pathways: lipid oxidation, oxylipin biosynthetic process, sexual reproduction, hydrolase activity and negative regulation of peptidase activity (Fig. 5A). Kyoto Encyclopedia of Genes and Genomes (KEGG) analysis showed that the DEGs were significantly enriched in metabolic pathways involved in amino sugar and nucleotide sugar metabolism, phenylpropanoid biosynthesis, starch and sucrose metabolism, and linoleic acid metabolism (Fig. 5B). Further analysis revealed that numerous genes associated with kernel development were downregulated in *zmmybr29*. As endosperm cells undergo cell proliferation and differentiation, the BETL and ESR regions are responsible for the transport of nutrients. In contrast, starchy endosperm serves as a nutrient storage site for kernel development and germination. Genes such as *Sugary2* (*Zm00001d037234*), *starch synthase I* (*Zm00001d045261*) and *starch phosphorylase1* (*Zm00001d034074*), which are involved in starch synthesis and carbohydrate metabolism, were downregulated in *zmmybr29* (Fig. 5C; Additional file 6: Table S5). Consequently, the abnormal nutrient transport mediated by BETL disrupts starch synthesis. Additionally, *ZmMYBR29* is involved in regulating of genes related to lipid synthesis and metabolism, as well as cell wall synthesis, which are part of biological pathways associated with kernel development (Additional file 1: Fig. S7C; Additional file 6: Table S5). Furthermore, DEGs related to fat synthesis and metabolism, cellulose synthase and cell wall regulatory proteins were also downregulated in *zmmybr29*. This suggests that the DEGs involved in cell expansion and dry matter accumulation during the filling stage were affected in *zmmybr29*.

### The expression of BETL-related genes was downregulated in *zmmybr29*

**Fig. 6:**
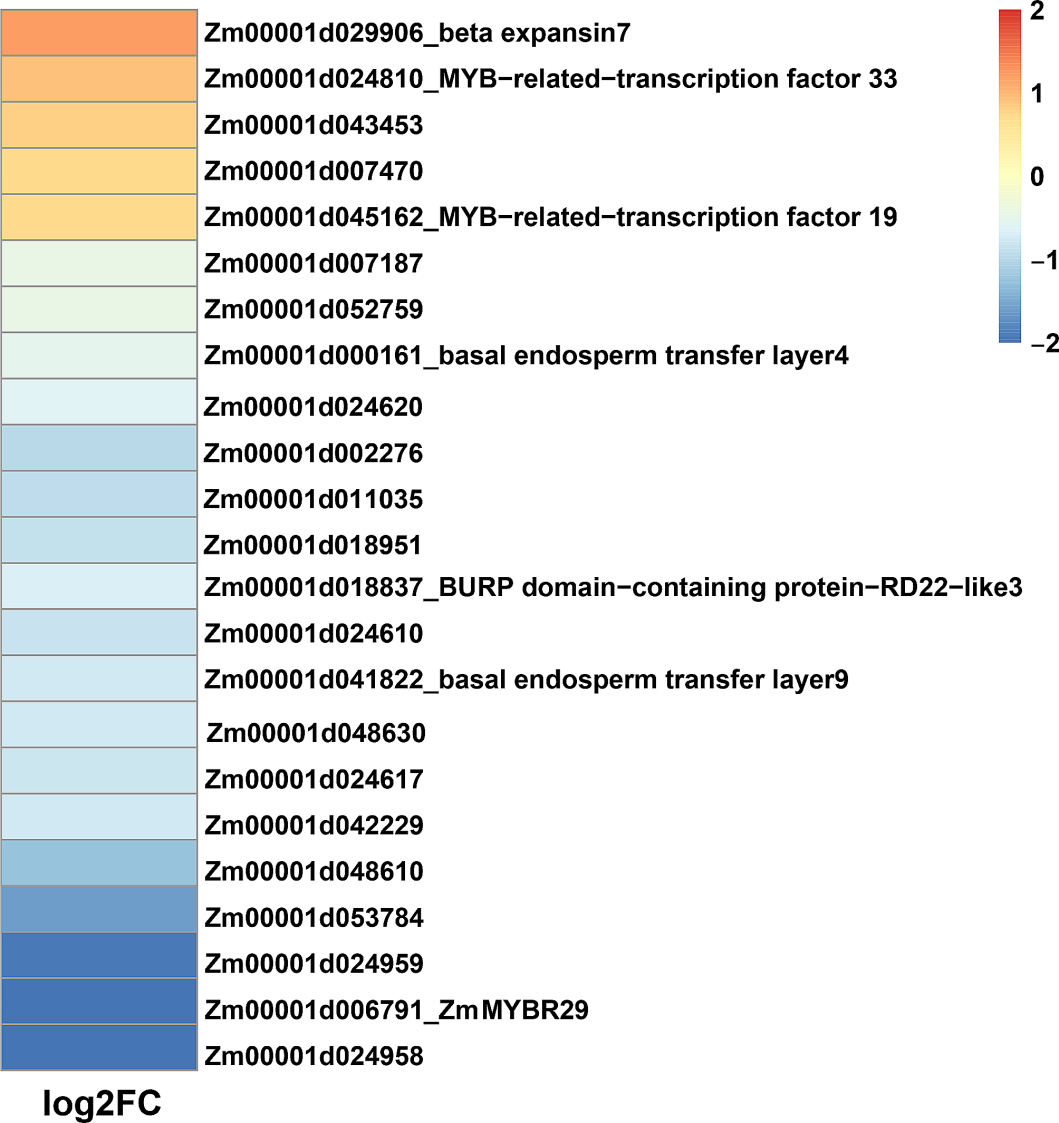
Heatmap of DEGs expressed specifically in the BETL from *zmmybr29* vs. WT. The heatmap depicts the log2 (Fold change) of genes selectively expressed specifically in BETL from *zmmybr29* vs. WT with blue to red colors indicating a transition from low to high fold change

According to the laser-capture microdissection data, a total of 23 DEGs were identified between the WT and *zmmybr29*, which expressed specifically in the BETL. Notably, the expression of *ZmMYBR29* was significantly downregulated in the mutant, indicating that the premature termination of translation also influenced its transcription level (Fig. 6). Among these genes, two members of the MYB transcription factor family, ZmMYBR19 and ZmMYBR33, exhibited elevated expression levels and displayed sequence homology with ZmMYBR29. These findings suggest that these two genes may function redundantly with *ZmMYBR29*. Additionally, the expression of BETL marker genes, *ZmBETL4* and *ZmBETL9*, which encode short peptides, were downregulated significantly. Nevertheless, the precise regulatory mechanisms underlying BETL development remain elusive.

Taken together, *zmmybr29* mutant exhibited altered expression patterns of genes associated with kernel development, particularly affecting the expression of BETL marker genes *ZmBETL4* and *ZmBETL9*, indicating that *ZmMYBR29* likely exert its effect by modulating the expression of these genes.

## Discussion

Through phylogenetic analysis, this study revealed that the transcription factor ZmMYBR29 belongs to the MYB-related transcription factor family (Fig. 1B). The results of in situ hybridization indicate that *ZmMYBR29* expressed specifically in BETL (Fig. 2A). We created a loss-of-function homozygous mutant by CRISPR/Cas9, and conducted statistical analysis on the traits of maize kernels of mature ears (Fig. 3D, E). The grain size of *zmmybr29* was smaller than the wild type, with the 100-grain weight decreased by 38% (Fig. 3F). These findings indicate that *ZmMYBR29* affects maize kernel size. Histological sections observation revealed reduced cell wall ingrowths of TCs and abnormal morphology of the BETL in *zmmybr29* mutant (Fig. 4A, B). The grain filling rate of *zmmybr29* mutant decreased by 26.7% compared to the WT (Fig. 4D). These results suggest that ZmMYBR29 participates in endosperm development by influencing BETL morphogenesis and function, thereby leading to reduced kernel size. Through Gene ontology analysis of RNA-seq data revealed altered expression level of genes related to starch synthesis, carbohydrate metabolism, cell wall synthesis and lipid metabolism involved in kernel development have been altered in *zmmybr29* (Fig. 5C; Additional file 1: Fig. S7C). This suggests that *ZmMYBR29* plays a regulatory role in kernel development and may significantly contribute to the storage accumulation phase.

In a recent report on the single-cell transcriptome of maize endosperm during cell differentiation, the research found that ZmMYBR29 is a pivotal factor in BETL development [23]. The single-cell transcriptomic analysis indicates a significant correlation between ZmMYBR29 and BETL-related cell clusters, and we have confirmed the result with additional evidence through in situ hybridization experiments, showing that *ZmMYBR29* expresses specifically in BETL (Fig. 2A). The recent research also demonstrates that the *mybr29* mutant exhibited reduced kernel size compared to the wild type. Microscopic examinations revealed abnormal TCs development and fewer cell wall ingrowths in *mybr29* mutant, which aligns with histological observations and statistical data, both of which prove that *ZmMYBR29* affects kernels size by participating in the TCs morphogenesis (Figs. 3D-F and 4A and B). We further demonstrated the influence of *ZmMYBR29* on BETL function by measuring the grain filling rate, which led to a decrease in grain weight (Fig. 4C, D). In summary, we have demonstrated that *ZmMYBR29* not only participates in the TCs morphogenesis, but also affects kernels size by influencing the material transport function of BETL.

The subcellular localization of ZmMYBR29 was observed in both the nucleus and cytoplasm of maize protoplasts, which is not typical of the nuclear localization of transcription factors (Additional file 1: Fig. S8). There are also many studies on the colocalization of transcription factors in both the nucleus and cytoplasm. The novel bHLH transcription factor KDR (KIDARI), which regulates the shade avoidance syndrome (SAS), is known to colocalize in both compartments. When KDR interacts with PAR1, PAR2 and other negative regulators of the SAS response, it is localized to the cytoplasm [44]. In *Arabidopsis*, the R1R2R3-MYB transcription factor MYB3R4, which responds to cytokinin and is localized in both the nucleus and cytoplasm, regulates cell division. High concentrations of cytokinin can promote the nuclear translocation of MYB3R4 through interaction with IMPORTIN ALPHA 3 (IMP A3), thereby regulating the expression of cell cycle-related genes and promoting the plant mitotic process [45]. BZR1 and BES1 are key transcription factors within the brassinosteroid (BR) signaling pathway. Under BR signaling induction, BZR1 and BES1 exhibit nuclear localization [46, 47]. It is speculated that ZmMYBR29 may be carried into nuclear by interacting with other proteins containing nuclear localization sequences (NLSs) and needs signal stimulation for its nuclear import. Further experimental verification is necessary to elucidate exactly how ZmMYBR29 works as a transcription factor.

The MYB-related transcription factor family, as a subfamily of MYB transcription factors, is widely present in plants. However, distinct from another subfamily, the R2R3-MYB, the MYB-related transcription factors have not been as extensively characterized [32]. Despite this, recent studies have begun to unravel the functions of the MYB-related transcription factors. OsMYBR57, a member of the MYB-related transcription factor family, has been shown to interact with the homeodomain transcription factor OsHB22 to regulate directly the expression of the drought-related *OsbZIPs*, thereby enhancing the drought tolerance of rice [35]. Ectopic overexpression of *OsTCL1* promoted the formation of trichome and root hairs [39]. TaMYB72 promoted flowering by upregulating the expression of florigen genes *Hd3a* and *RFT1* [40]. The MYB-related transcription factor bound directly to the core *cis*-elements of genes involved in stress response and plant development, there is a paucity of research exploring the role of MYB-related transcription factors in kernel development. In maize, ZmMRP1 (myb-related protein 1), which contains a MYB DNA binding domain, was the first BETL-specific transcription factor to be identified. When ZmMRP1 is activated by a promoter expressed specifically in AL, some of these cells transform into BETL cells with a typical sponge-like network structure, demonstrating that ZmMRP1 is a key regulatory factor for the differentiation and development of BETLs [41]. *ZmMRP1* expressed shortly after fertilization in the multinuclear coenocytic endosperm, with transcript accumulation in the basal domain destined to form transfer cells. After the completion of endosperm cell differentiation, cells in this region differentiate to form TCs. This indicates that the MYB-related transcription factor family potentially plays a role in regulating maize kernel development, making it worthy of further investigation. Several studies have shown that many BETL-specific genes such as *ZmBETL1*, *ZmBETL2*, *ZmBETL4*, *ZmBETL9*, *ZmBETL10*, *ZmMEG1*, and *ZmTCRR1* are directly regulated by ZmMRP1 [15, 18, 23, 41–43]. According to RNA-seq data analysis, *ZmBETL4* and *ZmBETL9* are downregulated in *zmmybr29* mutant (Fig. 6), indicating that they are also potential target genes of ZmMYBR29, which requires further experimental verification.

In this study, we used in situ hybridization to verify that *ZmMYBR29* expressed specifically in the BETL. Structural domain and protein sequence alignment revealed that ZmMYBR29 and ZmMRP1 have a high degree of amino acid sequence similarity, both of which contain a single SANT/Myb like conserved domain and belong to the MYB-related subfamily. The above results demonstrated a significant homology between ZmMYBR29 and ZmMRP1. Transcriptome data analysis revealed that the expression levels of 23 BETL-specific genes alter significantly in *zmmybr29*. Similar to ZmMRP1, ZmMYBR29 represents a potential MYB-related transcription factor influences grain filling and thereby affects maize kernel size.

## Conclusions

In summary, this study has investigated preliminarily the role of a MYB-related transcription factor ZmMYBR29, which expressed specifically in BETL, in the development of maize endosperm. This finding offers novel insights for further elucidating the molecular mechanisms underlying maize kernel size regulation, thereby advancing our understanding of this critical trait in crop improvement.

## Methods

### Plant materials

The wild-type material used in the maize (*Zea mays* L.) genetic transformation experiment via the *Agrobacterium*-mediated method was inbred line B104. The transgenic plants obtained from the T_0_ transgenic plants were planted in the greenhouse of Shandong Agricultural University in Tai’an, China, under simulated long-day lighting conditions and a temperature setting of approximately 28℃. The T_1_ and T_2_ transgenic plants (from which the CRISPR/Cas9 vector was isolated) were cultivated in the field in Tai’an, China and Hainan, China. All plants were self-pollinated.

Developing kernels were harvested from B104 and *zmmybr29-*1 ears from 4 to 30 DAP for statistical analysis of grain phenotype and grain filling rate (g/d) with three biological replicates. The grain filling rate = the difference in dry weight of 100 grains between two samples times/2. The material used for plant height statistics was cultivated in the field in Tai’an, China, 70 days after planting.

We carried out experiments with a stable, genetically modified T_2_ transgenic plants. T_0_ transgenic plants were acquired through *Agrobacterium*-mediated transformation of maize embryos, followed by a backcross with the inbred line B104. PCR analysis was used to identify plants with vector separation and genomic editing resulting in heterozygous knockout mutants among the T_I_ transgenic plants. The T_1_ heterozygous transgenic plants were allowed to self-cross, and subsequent PCR screening led to the selection of homozygous knockout mutants with smaller kernels in the T_2_ transgenic plants, which were then used for further experiments.

### CRISPR/Cas9 vector construction and transformation

To construct the loss-of-function mutant using the CRISPR/Cas9 system [48]. We analyzed the off-target efficiency using the CRISPR RGEN Tools website (http://www.rgenome.net/) and designed specific target sites on the first exon of *ZmMYBR29* (Gene ID: *Zm00001d00679*; Target-1:5’-AGTGTCTCCGAGATCAAGA-3’; Target-2: 5’-AAGAAGCACAGCGACATCG-3’). The primers used to construct the CRISPR/Cas9 vector (pBUE411 vector) are listed in Additional file 7: Table S6. Two transgenic lines of *ZmMYBR29* (*zmmybr29-*1, *zmmybr29-*2) were harvested for phenotypic analysis, and *zmmybr29-*1 labeled *zmmybr29* if not indicated otherwise was used for further experiments.

### Cytological analysis

To observe the cell morphology of maize endosperm. Developing kernels were harvested from the ears of B104 and *zmmybr29-*1 plants from 4 to 18 DAP. Each sample consisting of 10–15 kernels was cut along the longitudinal axis from the middle of 3 independent ears as a mixed pool. The slices containing the embryo were immediately placed in FAA fixative solution (formalin: acetic acid: ethanol = 1:1:10) on ice, vacuumed for 20 min at two times, and then fixed for 12–16 h at 4℃. The fixed material was dehydrated using a graded series ethanol for 1–3 h (70%,80%,90%,95%,100%), clarified with xylene for 30 min at three times and embedded in paraffin. The samples are cut into 8 μm slices using an RM2235 microtome (Leica, Wetzlar, Germany). After being deparaffinized and rehydrated, the samples were stained with 0.1% TBO (toluidine blue O) to observe the morphology of the kernels at different developmental stages. The samples for making semithin sections are cut into 2 μm slices using a diamond knife and were stained with 0.1% TBO. Images were acquired with an Olympus BX51 light microscope.

### Transmission electron microscopy

For sample preparation, mature 12 and 18 DAP kernels were removed from self-pollinated ears, and the bottoms were cut off with a razor blade and sliced into 1.2–2 mm sections, which were then fixed in 2.5% glutaraldehyde and 1% osmium tetroxide overnight at 4℃ [49]. After dehydration with a series of gradient ethanol solutions, the sample was transferred to propylene oxide and then embedded in acrylic resin. Make 2 μm slices using a diamond knife and observed with a transmission electron microscope (JEM-1400Plus) at Shandong Agricultural University.

### Phylogenetic analysis

To construct a phylogenetic tree, the full-length protein sequence of *ZmMYBR29* was used to search for homologous proteins in Arabidopsis, rice, sorghum, soybean, and wheat in the National Center for Biotechnology Information (NCBI, https://www.ncbi.nlm.nih.gov/) database. We performed sequence alignment using MEGA X. Then, we constructed a phylogenetic tree via the neighbor-joining method.

### In situ hybridization

In situ hybridization was performed on previously reported methods with some modifications. Briefly, to prepare the samples, 3–8 DAP kernels were removed from the middle of the ear, dehydrated, transparentized, and then embedded in paraffin to make 8 μm slices. The probe of ZmMYBR29 was synthesized using special cDNA as a template with T7 RNA Polymerase (cat. no. 10,881,767,001, Roche) and labeled with Digoxigenin-11-UTP (cat. no. 11,209,256,910, Roche). The primers synthesized for the *ZmMYBR29* probe are list in Additional file 7: Table S6. After dehydration, the slices were hybridized overnight with a labeled probe. The reaction was then carried out using AP (Anti-Digoxigenin-AP, Fab fragments, cat. no. 11,093,274,910, Roche), and finally reacted with NBT/BCIP Stock Solution (cat. no. 11,681,451,001, Roche). Images were acquired with an Olympus BX51 light microscope.

### RNA extraction and RNA-seq

For RNA-seq, three biological replicates of 12 DAP kernels were collected from six independent ears of B104 and *zmmybr29* plants. Total RNA was extracted with the Trizol reagent (cat. no. 15596-026, Ambion) and an Ultrapure RNA Kit (CWBIO, Beijing, China). Library construction and sequencing were completed by Lianchuan (Beijing, China). The sequencing platform used was an Illumina NovaSeq™ 6000 with PE150 and double-end sequencing mode. The six samples generated a total of 43.2 Gb of raw data. We evaluated the raw data after downloading. The Q20 and Q30 values were over 99% and 98%, respectively. After removing adapters, filtering low-quality reads and other preprocessing steps, we obtained a total of 42.43 Gb of effective data. The genes were analyzed for differential expression using DESeq2 software (fold change ≥ 2, FDR < 0.05), accompanied by the removal of genes with low expression levels (TPM < 1).

### Reverse-transcription quantitative PCR

Real-time PCR was performed on three biological replicates for each sample, using a SYBR Green qRT-PCR kit (TIANGEN) according to the manufacturer’s instructions on a Light Cycler 96 (Roche Diagnostics). The maize Actin gene (*Zm00001d010159*) was used as the internal control. The primers used in this study are listed in Additional file 7: Table S6.

### Subcellular localization

The full-length open reading frame (ORF) of *ZmMYBR29* was cloned and inserted into the expression vector PM999-green fluorescent protein (GFP). The primers used to amplify *ZmMYBR29* are listed in Additional file 7: Table S6. To ensure the transformation efficiency of the protoplasts, we used the Plasmid Maxprep Kit (cat. no. N001, Vigorous Biotechnology) to extract the plasmids to ensure the concentration of the plasmids. The methods used for the preparation and transformation of protoplasts previous reports [50]. Briefly, protoplast were extracted from the etiolated seedlings at 10 DAG. After morphological detection under a light microscope, when more than 70% of the protoplasts were intact, transformation continued. After dark treatment with PEG solution for 13 min, overnight culture in the ark was performed for 12 h. The photo was captured with an LSM 880 microscope.

### Electronic supplementary material

Below is the link to the electronic supplementary material.

Supplementary Material 1

Supplementary Material 2

Supplementary Material 3

Supplementary Material 4

Supplementary Material 5

Supplementary Material 6

Supplementary Material 7

## Data Availability

The dataset supporting the conclusions of this article is available in the NCBI Sequence Read Archive (SRA) platform under the accession number PRJNA1077719 (https://www.ncbi.nlm.nih.gov/bioproject/PRJNA1077719/).

## Abbreviations

TCs: Transfer cells
BETL: Basal endosperm transfer layer
TEM: Transmission electron microscope
DAP: Days after pollination
AL: Aleurone layer
ESR: Embryo surrounding region
SE: Starchy endosperm
CWIs: Cell wall ingrowths
PC: Placento-chalazal
DEGs: Differentially expressed genes
GO: Gene ontology
KEGG: Kyoto Encyclopedia of Genes and Genomes
