# Supplementary material for: A MYB-related transcription factor ZmMYBR29 is involved in grain filling
Source: BMC Plant Biol. 2024 May 27;24:458. doi: 10.1186/s12870-024-05163-9 (PMC11129368; doi:10.1186/s12870-024-05163-9)
Supplement: Supplementary file 1 — Supplementary Material 1 [file 12870_2024_5163_MOESM1_ESM.docx]

**Fig. S
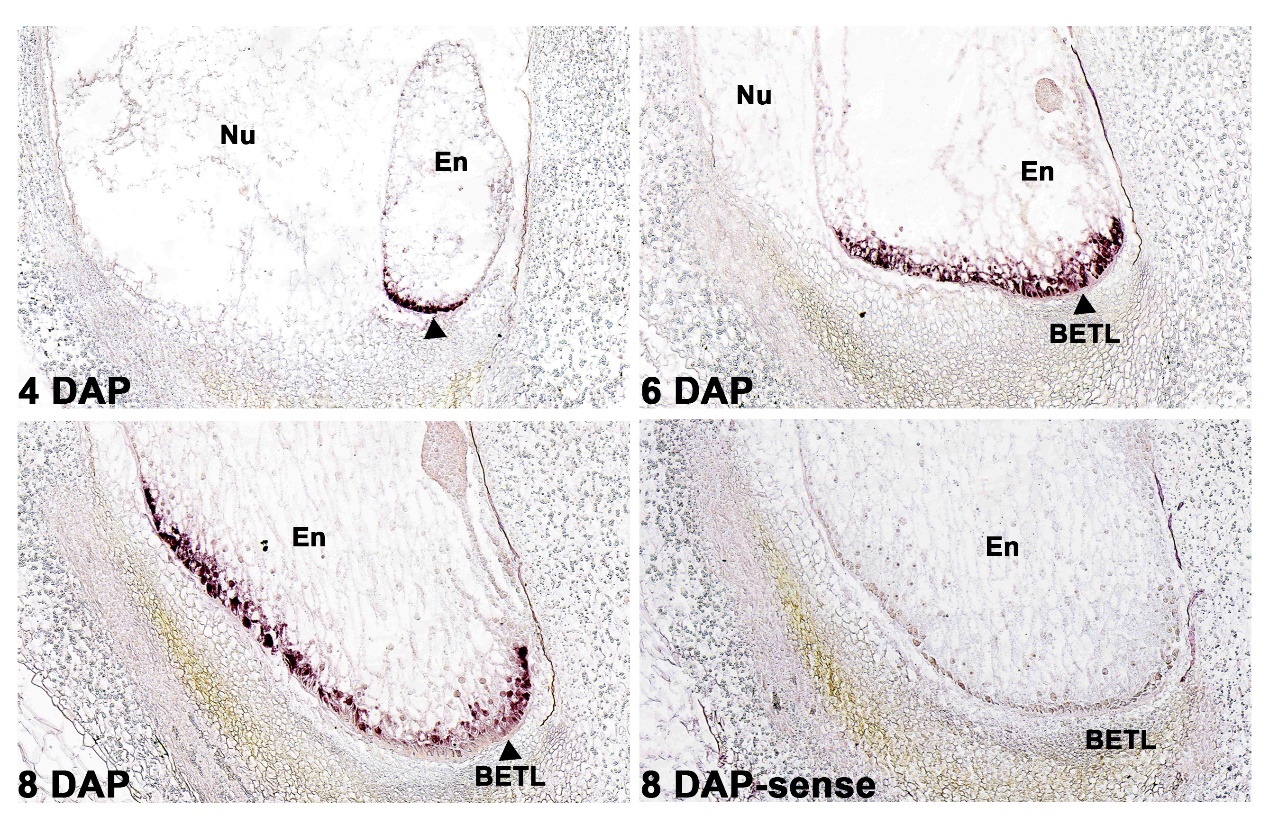
1** In situ hybridization analysis of the expression of *ZmMYBR29*. Histological sections of maize kernels at 4, 6 and 8 DAP were hybridized with antisense and sense. *ZmMYBR29* was expressed at the basal of endosperm, as indicated by the black arrow. Nu, nucellus; En, endosperm; BETL, basal endosperm transfer layer.

**
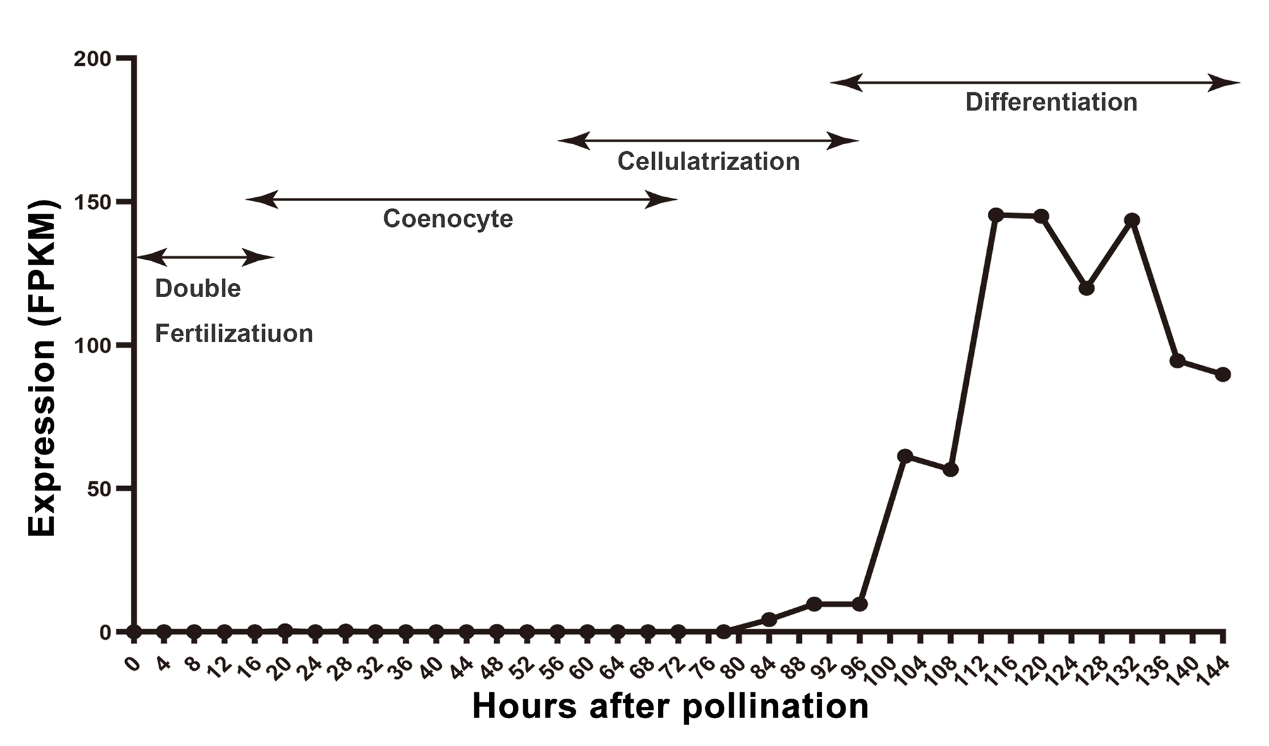
Fig. S2** The expression of *ZmMYBR29* in the early development stage of maize according to data analysis. Analysis of the expression level of *ZmMYBR29* in the early developmental stages of maize kernels from 0 to 144 hours after pollination.


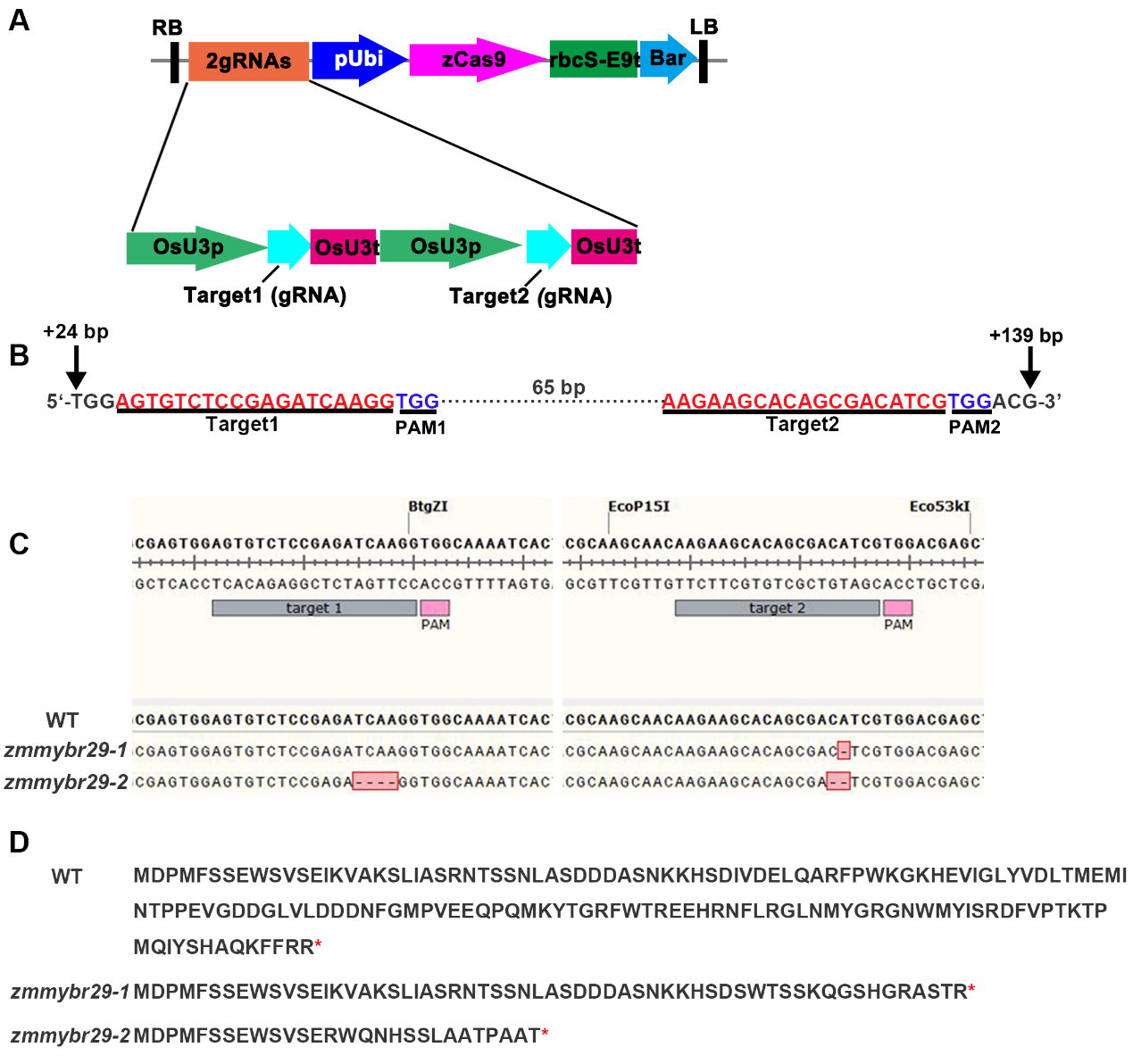
**Fig. S3** Construction of CRISPR/Cas9 vector for *ZmMYBR29*. **A** Schematic diagram of CRISPR/Cas9 vector. **B** Target sequence. The blue words indicate PUM sites, the red words indicate target DNA, and the black arrow indicates the position of the base sequence on the coding sequences (CDS). **C** Identification of gene editing plants by sequencing. The red box represents base deletion. **D** The protein sequences of two loss-of-function transgenic lines. The red asterisk indicates that the translation was terminated.


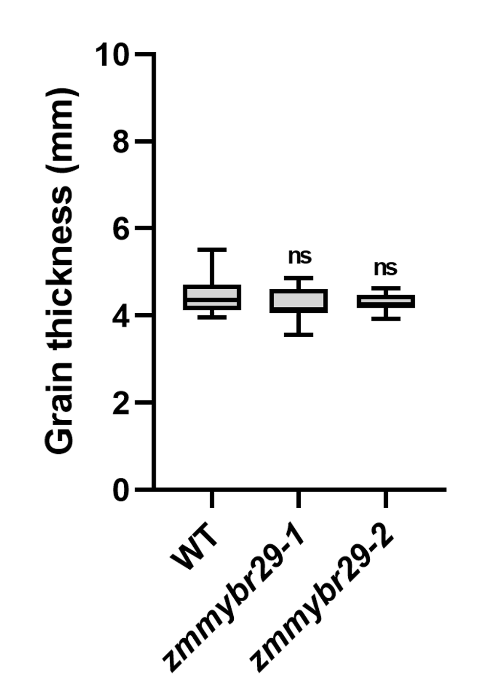
**Fig. S4** Grain thickness analysis of WT and *zmmybr29*. Grain thickness analysis of individual mature seeds from the T_2_ homozygous transgenic plants. ns, no significance (Student’s *t*-test).

**
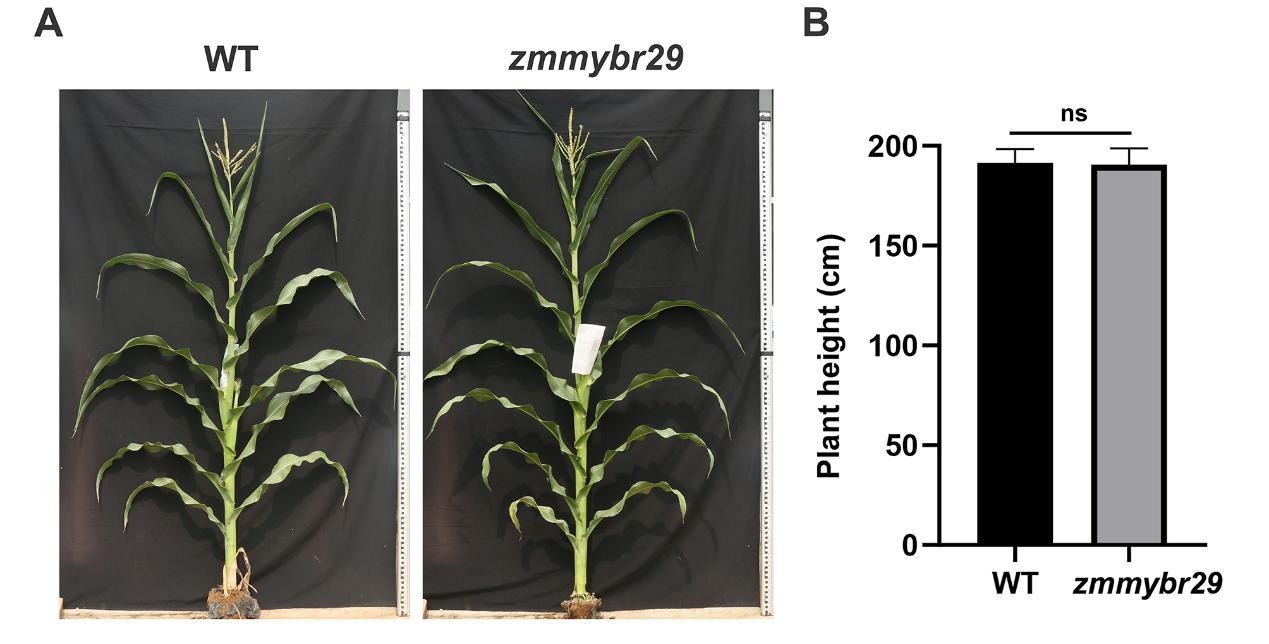
Fig. S5** Phenotypes of WT and *zmmybr29* plants. **A** Comparison of mature plants height. The left picture shows WT and the right picture shows *zmmybr29*. **B** Cartogram of plant height. The black box represents WT, the gray box represents *zmmybr29*. Error bars indicate SD. ns, no significance (Student’s *t*-test).


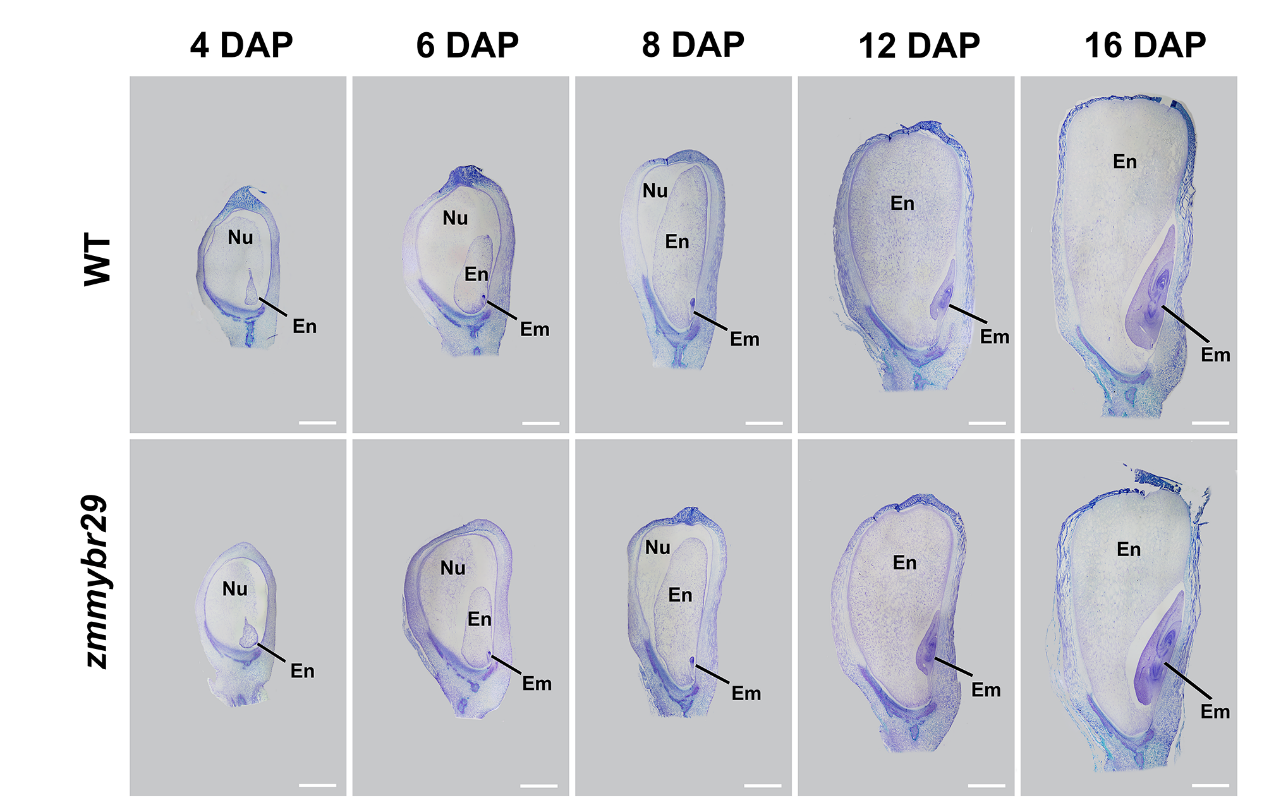
**Fig. S6** Histological sections of WT and *zmmybr29* kernels. Histological sections stained with 0.1% TBO of WT and *zmmybr29* kernels of 4 DAP-16 DAP. Nu, nucellus; En, endosperm; Em, embryo; Scale bars, 1 mm.


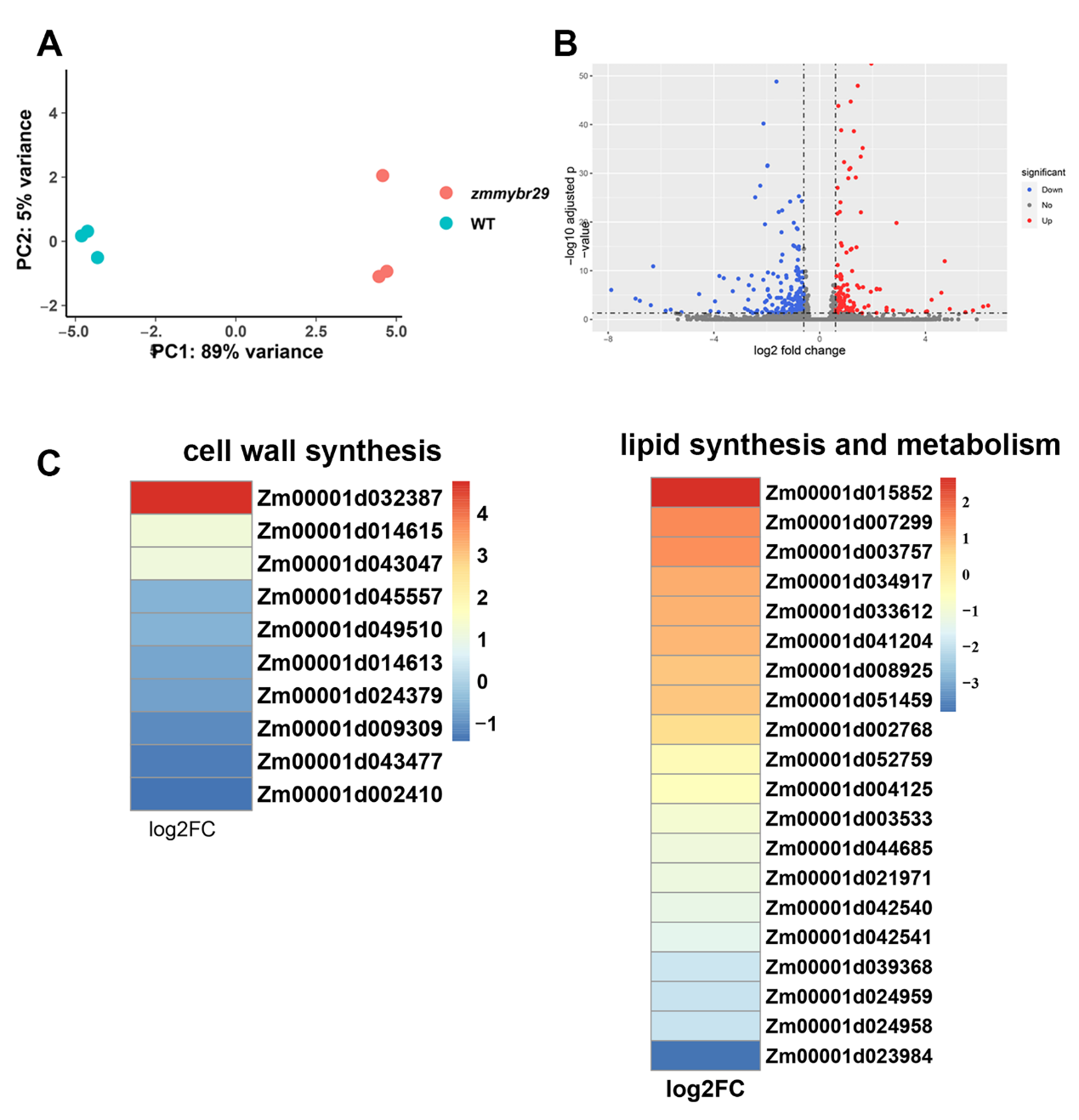
**Fig. S7** Transcriptome sequencing data analysis. **A** Principal component analysis diagram. Different colors represent different samples. **B** Volcano map of DEGs. The abscissa represents the logarithm value of the multiple difference of gene expression level between the two samples. The ordinate represents the negative logarithm value of FDR. **C** Gene expression heatmap related to cell wall synthesis, lipid synthesis and metabolism.

**Fig. S**
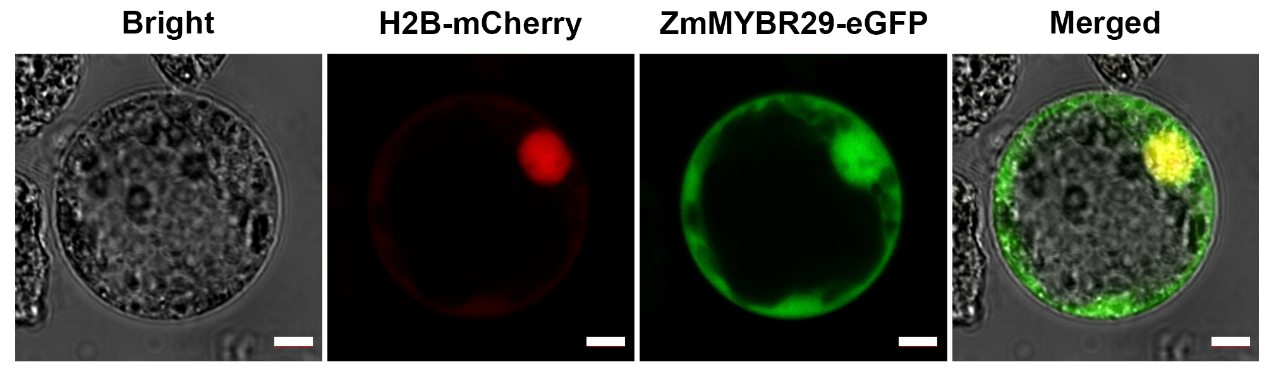
**8** Subcellular Localization of ZmMYBR29. Detecting the subcellular localization of ZmMYBR29 in dissociated protoplasts from 10 day after planting maize seedlings with H2B as the nuclear marker gene. Scale bars, 5 μm.
